# Investigating the prevalence of pathogenic variants in Saudi Arabian patients with familial cancer using a multigene next generation sequencing panel

**DOI:** 10.18632/oncotarget.28457

**Published:** 2023-06-12

**Authors:** Musa AlHarbi, Nahla Ali Mobark, Wael Abdel Rahman AlJabarat, Hadeel ElBardis, Ebtehal AlSolme, Abdullah Bany Hamdan, Ali H. AlFakeeh, Fatimah AlMushawah, Fawz AlHarthi, Abdullah A. AlSharm, Ali Abdullah O. Balbaid, Naji AlJohani, Alicia Y. Zhou, Heather A. Robinson, Saleh A. Alqahtani, Malak Abedalthagafi

**Affiliations:** ^1^Department of Pediatric Oncology, Comprehensive Cancer Centre, King Fahad Medical City, Riyadh, Saudi Arabia; ^2^Genomics Research Department, King Fahad Medical City, Riyadh, Saudi Arabia; ^3^Department of Oncology, King Fahad Medical City, Riyadh, Saudi Arabia; ^4^Department of Surgery, King Fahad Medical City, Riyadh, Saudi Arabia; ^5^Department of Medicine, King Fahad Medical City, Riyadh, Saudi Arabia; ^6^Color Health Inc., Burlingame, CA 94010, USA; ^7^Health eResearch Centre, University of Manchester, Manchester, UK; ^8^Division of Gastroenterology and Hepatology, Johns Hopkins University, Baltimore, MD 21287, USA; ^9^Liver Transplant Center, King Faisal Specialist Hospital and Research Center, Riyadh, Saudi Arabia; ^10^Department of Pathology and Laboratory Medicine, Emory University Hospital, Atlanta, GA 30322, USA

**Keywords:** cancer, hereditary cancer syndrome, genetic counseling, NGS, cancer screening

## Abstract

Family history is an important factor in determining hereditary cancer risk for many cancer types. The emergence of next-generation sequencing (NGS) has expedited the discovery of many hereditary cancer susceptibility genes and the development of rapid, affordable testing kits. Here, a 30-gene targeted NGS panel for hereditary cancer risk assessment was tested and validated in a Saudi Arabian population. A total of 310 subjects were screened, including 57 non-cancer patients, 110 index patients with cancer and 143 of the cancer patients’ family members, 16 of which also had cancer. Of the 310 subjects, 119 (38.4%) were carriers of pathogenic or likely pathogenic variants (PVs) affecting one or more of the following genes: *TP53, ATM, CHEK2, CDH1, CDKN2A, BRCA1, BRCA2, PALB2, BRIP1, RAD51D, APC, MLH1, MSH2, MSH6, PMS2, PTEN, NBN/NBS1* and *MUTYH*. Among 126 patients and relatives with a history of cancer, 49 (38.9%) were carriers of PVs or likely PVs. Two variants in particular were significantly associated with the occurrence of a specific cancer in this population (APC c.3920T>A – colorectal cancer/Lynch syndrome (*p* = 0.026); *TP53* c.868C>T; – multiple colon polyposis (*p* = 0.048)). Diverse variants in *BRCA2,* the majority of which have not previously been reported as pathogenic, were found at higher frequency in those with a history of cancer than in the general patient population. There was a higher background prevalence of genetic variants linked to familial cancers in this cohort than expected based on prevalence in other populations.

## INTRODUCTION

Familial/hereditary cancer syndromes (FCS) are an important component of overall cancer incidence, therefore family history of cancer is a key factor in determining overall cancer risk and prognosis [1–3]. FCS is of particular concern in Saudi Arabia, which has one of the highest rates of consanguinity worldwide [4–7]. Cancer increased in the Kingdom of Saudi Arabia by 136% between 1999 and 2015 [4].

An estimated 20% of all cancer patients in Saudi Arabia have a positive family history of cancer [5–8] and are therefore likely to carry mutant alleles. Where a mutation is carried in one allele, damage to the second, wild type allele at the same locus can result in the loss of a checkpoint to cancer development [9]. The clustering of cancer in families may be due to shared environmental exposures and/or inherited genetic factors, including complex interactions between the two; hereditary causes alone account for ~5% of regional cancer cases [8].

The most common forms of cancer in the population are breast and colorectal cancer, with respective prevalences of 53% and 51% [10], both of which are associated with FCS. Although breast cancer incidence is lower than in most Western populations, it is steadily climbing, and is expected to eventually plateau at an ASR comparable to Western countries as lifestyles change over time [11]. Age at onset is lower than is typical globally, and women are likely to present at a later stage, with poorer prognoses.

Colorectal cancer incidence in Saudi Arabia was estimated at 14.4% in 2020, with a higher than typical level of early onset CRC [12]. A 2020 study of colorectal cancer (CRC) in Saudi Arabia found that colon cancer patients with a corresponding family history had a statistically significant higher risk of mortality than colon cancer patients with no family history of CRC [5]. CRC can be familial when caused by familiar adenomatous polyposis (FAP), or by Lynch syndrome, which appears to be more common in this population than in Western countries [12].

As the prevalence of FCS and cancer-related mortality in the Saudi population appears to be increasing [6, 7, 13–18], an understanding of which pathogenic variants (PVs) are prevalent in the Saudi population has practical value. As approximately 40% of all marriages in Saudi Arabia are between relatives [19, 20], it is important to inform related individuals who wish to marry about the risk to future offspring of inheriting cancer-associated alleles from both parents. Moreover, knowing which variants in which genes contribute to the cancer incidence in Saudi populations would allow both for genetic screening and for physicians to recommend preventative measures, including surgical interventions or simply regular physical or radiological screening, to those at high risk due to inheritance of mutations. It could also be appropriate for relatives who have not inherited variants associated with familial cancer to reduce their frequency of screening via procedures such as colonoscopy or mammography, which have associated medical risks [2, 10]. However, to reap these benefits, the exact variant(s) being transmitted in the family must be known, and this can require screening multiple genes to find relevant PVs.

Recent advances in the field of medical genomics and, in particular in the use of next-generation sequencing (NGS), have opened new avenues for a better understanding of the underlying genetic risk factors for cancer [13–21]. NGS gene panels allow for the screening of multiple cancer-associated genes simultaneously, making them a cost-effective and time-saving way to detect familial variants [13, 16]. In particular, they detect inherited mutations in individuals who might not meet the criteria of international screening standards, and, in individuals who have inherited a familial cancer-associated mutation in one gene, multi-gene panels detect variants in additional genes that may increase the cancer risk for those individuals [16, 22]. Furthermore, using an NGS gene panel approach has been shown to detect cancer-associated germline mutations in genes not generally associated with the type of cancer that runs in the family, which would have been missed if only the genes associated with the particular cancer type had been sequenced [16].

There are many studies which have screened for germline mutations in populations with few or no Arab individuals [16, 23]. However, since the prevalence of individual mutations may vary across populations, it is important to determine the prevalence of cancer-associated mutations within specific populations [14, 24, 25]. Previous studies using gene panels to screen for cancer-associated mutations have typically involved either large but non-Arab-specific populations [15, 23, 26], or Arab-specific but small-sized populations [14].

Studies specific to Arab populations have typically screened for tumor-specific rather than broad germline mutations [14, 15, 26, 27], or have focused on one gene or a small number of genes associated with one type of cancer [28–44]. These show that Arab cohorts differ greatly in pathogenic variant prevalence from Western cohorts, and from each other. A meta-analysis of ovarian cancer cohorts across 22 Arab-predominant countries, has revealed eight ovarian-cancer linked mutations apparently unique to Arab populations, six of which were only observed in Saudi Arabia, with the most commonly observed mutations being in BRCA1 (77% of patients) [45].

In a Lebanese study, fewer than 6% of patients with early onset or familial breast cancer carried a known *BRCA1* or *BRCA2* PV, suggesting that alternative variants drive breast cancer incidence in this population. A total of 12% carried a VUS, with 26% having one haplotype featuring the same seven variants, and the haplotypes observed in Lebanese patients varied significantly from those observed in Tunisian and Algerian patients [46].

Two previous case studies have demonstrated links between ‘rare’ variants and familial cancer in Arab populations. In the first, the extremely rare *TP53* missense variant, c.799C > T (p. Arg267Trp) was found in a 2-year-old Saudi proband diagnosed with choroid plexus carcinoma (CPC), and six first- and second-degree relatives [47]. This family comprised seven of the eight known individuals with this mutation in the Saudi population, six of which will be analyzed in the current study. The authors also observed this variant in an additional, unrelated individual with colon, breast and ovarian cancer, indicating the potential relevance of this variant to multiple types of cancer in this population. A second study reports on a 5-year-old female, whose data is also included in this study, with glioblastoma multiforme and constitutional mismatch repair-deficiency due to an *MSH6* homozygous c.1883G>A mutation, who continued to experience an exceptional and durable response, 9 months later, to the immune checkpoint inhibitor (ICPI) nivolumab [48]. To the authors’ knowledge, this was the first report in the Arab world of a durable response to ICPIs in a pediatric glioblastoma patient. There is minimal other literature on specific cancer-related genetic variants in the Saudi population, which reinforces the importance of the current study.

In this study, candidates were screened using a commercial NGS panel kit comprising 30 whole or partial gene loci, for which variants have previously been reportedly associated with the development of breast, ovarian, colorectal, melanoma, pancreatic, prostate, uterine and stomach cancers based on the existing literature. This kit has been previously trialed as a means of capturing potential PVs at a population level in Nigeria and the Caribbean, and in identifying rare variants in cancer patients who have tested negative for common cancer variants [35–38]. Our results not only have an immediate clinical impact for the patients screened, but also provide important knowledge about population-specific genetic cancer risk factors that can guide future interventional programs, to prevent the development or progression of familial cancers, in Saudi Arabia and in Arab populations around the world.

## RESULTS

A total of 313 subjects were eligible for screening for sequence variants in 30 genes. For three of these patients, sequencing failed. The results of two patients had previously been recorded in another study, avoiding retesting. Within the remaining cohort, there were 310 patients and relatives (188 males and 122 females), with a mean age of 35.0 yrs (sd = 15.6 yrs), ranging from 6 months to 75 years (Supplementary Table 1). The mean age at cancer diagnosis (*n* = 32), was 39.1 ± 12.0 yrs.

**Figure 1:**
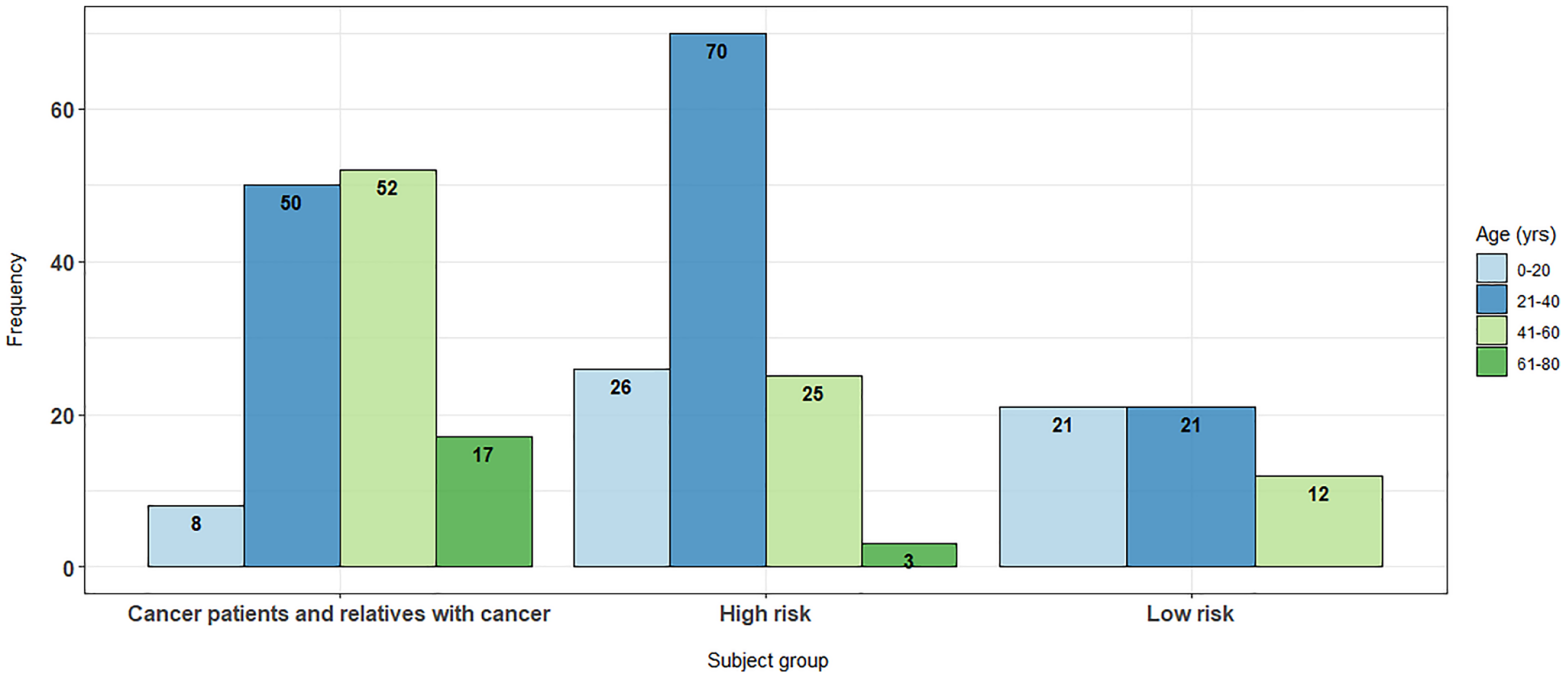
Age distributions across cohort subgroups.

We defined three subgroups of participants among the 313 subjects; these included 110 index patients with cancer, 143 of their family members, and 57 individuals without cancer. This latter group was designated low-risk for the purposes of the analysis, based on having no family history of cancer. The majority of these individuals were patients of the hospital for non-cancerous conditions, which justified their testing (Table 1, Figure 1).

**Table 1:** Subject characteristics

	*N*	%
Relationship to Proband^*^		
Self	110	35.48
Mother	20	6.45
Father	15	4.52
Son	18	5.81
Daughter	18	5.81
Sister	33	10.6
Brother	19	6.13
Granddaughter	3	0.97
Grandson	2	0.65
Maternal Aunt	1	0.32
Maternal Uncle	1	0.32
Paternal Aunt	3	0.97
Paternal Uncle	2	0.65
Nephew	2	0.65
Niece	5	1.61
Cousin	1	0.32
No family history of cancer	57	18.39
**Gender**		
Male	188	60.65%
Female	122	39.35%
**History of cancer**		
Yes	126	40.65%
No	184	59.35%
**Age (yrs)**		
<20	55	17.74%
20–39	141	45.48%
40–60	89	28.71%
>60	20	6.45%
Missing	5	1.61%
**Total**		
All subjects	310	100%

^*^Where patients are both index patients and relatives of other index patients, they are listed here as index patients.

Sixteen of the 143 family members of cancer patients (11.2%) had also been diagnosed with cancer at any time prior to this study, and are therefore grouped with the ‘index’ cancer patients for the purpose of the analysis. These diagnosed relatives and our index cancer patients are collectively referred to within the analysis as ‘persons with a history of cancer’.

Family members of cancer patients, who had never prior to this study been diagnosed with any cancer, are designated separately within the analyses as ‘high risk’ individuals. We consider them to be at higher risk of developing cancers in the future than individuals with no family history of cancer.

The relatives of index patients tended to be their descendants, therefore the age distributions of patients varied significantly between these groups, with high-risk individuals and non-cancer patients both typically being younger than the index cancer patients (Figure 1; *p* < 0.001). Due to ethical considerations and the relatedness of the subjects it was not feasible to resample by age or to prospectively recruit to address the age skew.

Across all groups, there were more female than male participants, although the distribution of gender was similar across groups (*p* > 0.05). Information on consanguinity was only available for 27 of the patients, 12 of which reported consanguinity.

### Pathogenic variants

We identified germline PVs and likely PVs in the following genes: *APC, ATM, BRCA1, BRCA2, BRIP1, CDKN2A* (in both p14ARF and p16INK open reading frames), *CHEK2, MLH1, MSH2, MSH6, MUTYH, PMS2, PTEN*, and *TP53* (Supplementary Tables 2 and 3). Variants of undetermined significance (VUS) were observed in the following genes: *BRCA2, APC, ATM, PALB2, MSH2, MLH1, MSH6, CHEK2, CDKN2A, TP53, NBN, PMS2, RAD51D, BRIP1, BMPR1A,* and *CDH1*.

Roughly half of the 123 unique variants observed were singleton variants (*n* = 67, Supplementary Table 3). However, we identified 13 frequent variants occurring in five or more subjects, 2 of which are considered likely PVs (*APC* c.3920T>A and *TP53* c.799C>T), and six of which have been previously classified as PVs (*BRCA1* c.5123C>A, *MSH2* c.1964del, *MUTYH* c.544C>T and c.734G>A, *PMS2* c.1376C>G and c.1606C>T). *PMS2* c.1376C>G was the most frequent Lynch syndrome-associated variant in a survey of Saudi CRC patients [39].

**Figure 2:**
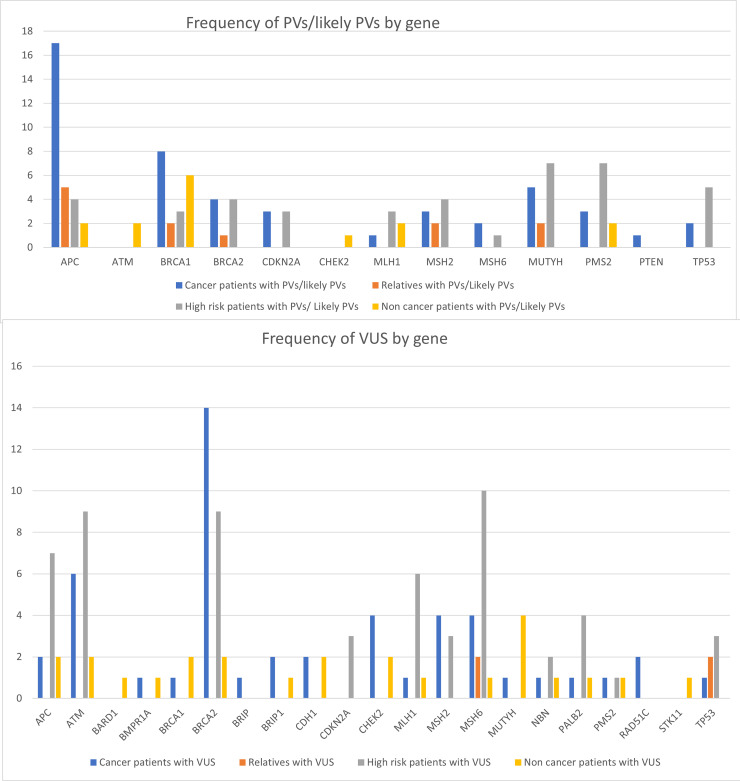
Frequencies of PVs and likely PVs per gene, by subject group. A list of all variants discovered in each patient group, along with any cancer types diagnosed in patients with these variants, can be found in the Supplementary Tables 1 and 4.

The proportions of persons with a history of cancer (including index patients and diagnosed relatives), high-risk participants (undiagnosed relatives of index patients) and low-risk participants (non-cancer patients with no patient history), with PVs, likely PVs and VUS, were determined and compared (Table 2, Figure 2). In total, there were 123 individual genetic variants found or reported among the 310 subjects (Supplementary Table 3), including 34 PVs, 7 likely PVs and 82 VUS. Considering the numbers of patients with and without PVs, likely PVs and VUS, there was no significant difference in the numbers of subjects with and without PVs (*p* > 0.05, Fisher’s exact test). Similarly, there was no significant difference in the proportion of patients with likely PVs across groups, or in the prevalence of VUS between groups (*p* > 0.05). However, the likelihood of homozygosity at one or more PV or likely PV was significantly higher in persons with cancer (*n* = 10 of 126, 7.9%) based on an exact test for a multinomial distribution (p LLR<0.05) when compared to one case of homozygosity at a PV/likely PV among the low-risk participants and only one case among the high-risk participants.

**Table 2:** Individuals with PVs, likely PVs and/or VUS

Number of patients with different variant classifications	Cancer patients (*n* = 110) and family members with cancer (*n* = 16) *n* = 126 (%)	High Risk Individuals *n* = 125 (%)	Low Risk Individuals *n* = 57 (%)
Pathogenic variant(s) (PVs)	36 (28.3)	31 (24.8)	12 (21.1)
Likely PV(s), no PVs	13 (10.3)	20 (16.0)	5 (8.8)
VUS only	29 (23.0)	26 (20.8)	17 (29.8)
Negative for all tested variants	48 (38.1)	48 (38.4)	21 (36.8)
Not tested- syndicated data	0	0	2 (3.5)

Based on a likelihood ratio exact test for a multinomial distribution, the frequencies of PVs and likely PVs in each subgroup indicate a statistically proportionate distribution.

There were 13 instances in which patients were homozygous at SNP loci, ten of which involved index cancer patients. Nine of these loci were PVs and three were likely PVs (Supplementary Tables 2, 3 and Supplementary Figures). There was one instance of a duplication in *MSH6* in a colon cancer patient, and three patients with duplications of *BRCA2* c.5557 within the same family, two of which reported breast cancer. Fifteen subjects had deletions within single exons, and seven patients had deletions spanning multiple exons in *BRCA1, APC, ATM, CHEK2* or *MSH2.* We observed a large chromosomal rearrangement of chromosome 9 removing *CDKN2A* (p.14ARF) in a bone marrow cancer patient (Supplementary Tables 1 and 3), and six out of 18 unique indel events were observed in multiple members of a family.

### Genes of interest

PVs in the *APC* gene were significantly more frequent in cancer patients than in the other groups and were observed at least twice as frequently in cancer patients than PVs in any other gene within the panel (p LLR = 0.0003). Other PVs appeared to be normally distributed (*p* > 0.05, Figure 2), although the overall numbers of subjects with most PVs were small enough that a likelihood ratio version of the exact test for a multinomial had to be used, so it is not surprising that no association was detected. *BRCA2* variants were also significantly more frequent in persons with cancer (*p* = 9.82e^−08^), with a notable enrichment of *BRCA2* VUS in these patients (Figure 2). We anticipate that some of the VUS flagged within this panel may prove pathogenic or to be linked to PVs if investigated in larger Arab population samples.

### Associations with cancer

Despite their approximately proportionate overall distribution, the various PVs do not have the same impact across all types of cancer, so it is important to consider the association of each individual PV with the various types of cancer observed. In order to detect any associations between individual variants and cancer occurrence, the observed cancers were grouped by the affected organ (or organ system). *APC* PVs/likely PVs were found in 20 of the 126 participants with cancer (15.9%). The most common types of cancer observed in patients with APC PVs/ likely PVs were colon cancer (*n* = 9), rectal cancer (*n* = 5) and breast cancer (*n* = 4). APC PVs/ likely PVs were also observed in 21 of 125 high-risk patients (16.8%) and 7 of 57 ‘low-risk’ participants with no family history of cancer (12.3%). The most common *APC* variant was likely PV c.3920T>A, occurring in 39 subjects, this was observed with *MSH2* Val655Aspfs*30 in 5 subjects, all from one family, and with *MSH6* variants in 7 cases, which again were all from the same family. There was a significant association of the *APC* c.3920T>A; p.Ile1307Lys variant with colorectal cancers, i.e. colon, rectal, and sigmoid cancers, as well as polyposis (*p*-value = 0.03).

Of the 19 persons with cancer carrying *BRCA2* variants, eight had breast cancer, seven of which lacked *BRCA1* variants. Four patients carrying *BRCA2* variants had rectal cancer, and three had colon cancer. All PVs observed within *BRCA2* were deletion or duplication events which significantly disrupt gene function (c.3170_3174del, c.4787del, c.5557dup), these appeared only in cancer patients and high-risk patients, with 5 of 7 *BRCA2* PV carriers affected. Carriers were significantly more likely to have breast cancer than non-carriers (*p* = 8e^−06^), with 50% of carriers having breast cancer across four unrelated families (Supplementary Table 3). The most frequent *BRCA2* variant in this sample was the previously considered VUS c.122C>T, although carriers with cancer did not report breast cancer. No individual *BRCA1* variants were significantly associated with cancer or breast cancer specifically within this sample, most likely due to sample size.

Other statistically significant associations included an association of *TP53* c.868C>T; p.Arg290Cys with multiple colon polyposis in this population. There were also 21 less common variants appearing 3 or more times whose risk association with cancer could not be quantified because they only appeared in cancer and/or high-risk subjects with a family history of cancer, and many unique variants (Supplementary Table 3). For example, there were 13 subjects with *MSH6* c.733A>T, which is considered a VUS, all of whom had cancer or were high-risk subjects. These frequent variants, unique to cancer and high-risk patients, which should be noted as potential PVs, included *TP53* c.799C>T, *APC* c.3183_3187delACAAA, *ATM* c.1516G>T, *BRCA2* c.3170_3174del, c.122C>T, c.5909C>T and c.5557dup, *CDKN2A* c.238C>T, *MSH2* c.1964del, *MSH6* c.733A>T deletion of the whole of exon 16 of *MLH1, MUTYH* c.544C>T and c.734G>A, and *PALB2* c.1102A>T.

**Figure 3:**
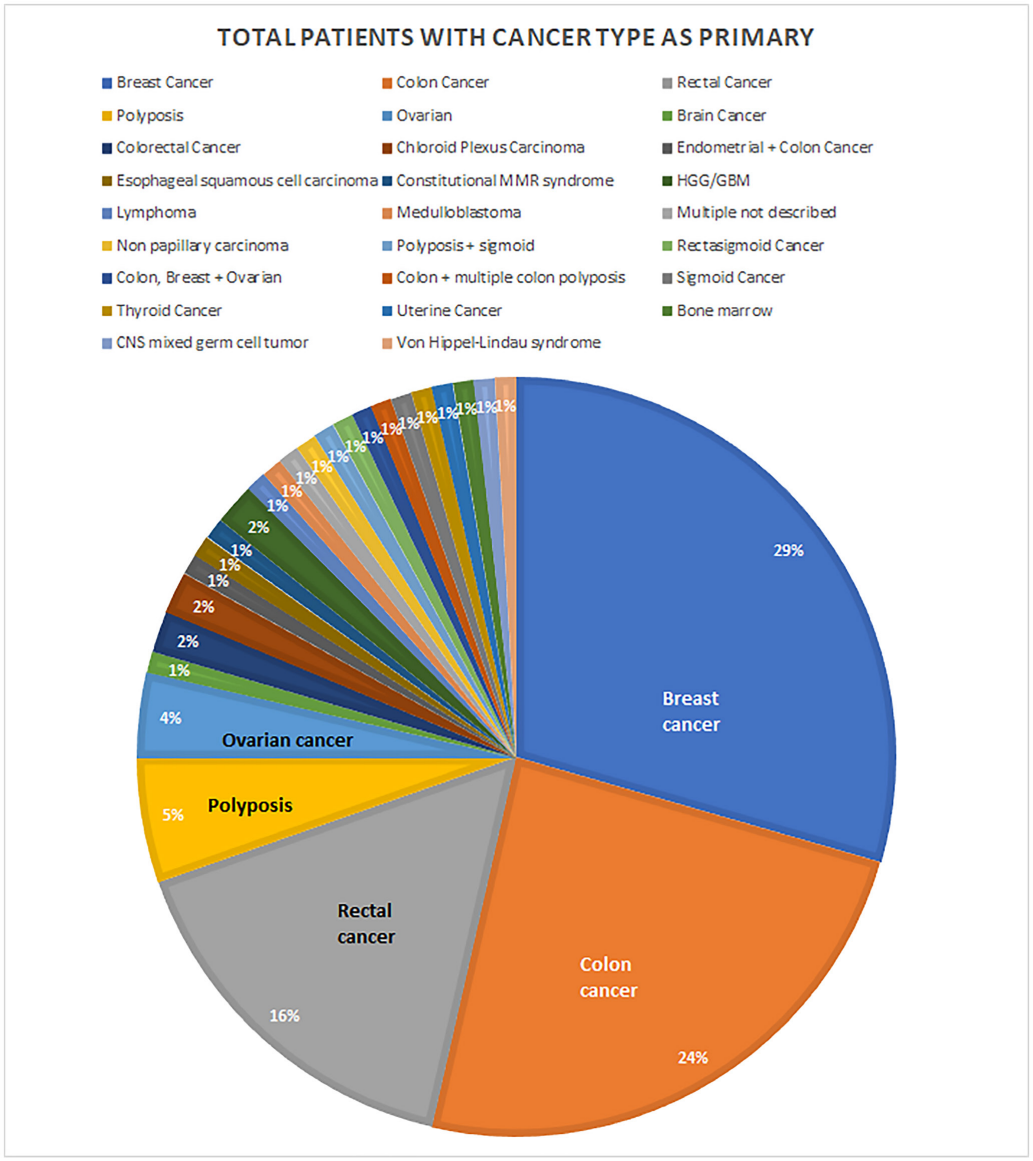
Locations of primary cancers. Some patients have reported multiple cancer types on primary presentation, each patient is counted once.

For persons with cancer, eight cancer types were most commonly observed. The most common primary cancer types were colorectal cancers, including colon, rectal, and sigmoid cancers, as well as polyposis (*n* = 60; 47.2% of persons with cancer), breast cancers (*n* = 34; 27.6%), and ovarian cancer (*n* = 5; 3.9%) (Figure 3). Some patients had more than one of these eight cancer types on primary presentation or had a different secondary cancer type (Supplementary Data 1).

A total of 107 participants, 49 (45.8%) of which had a history of cancer, had one or more PV or likely PV. Out of the remaining 203 participants, who were negative for the PVs and likely PVs included in the panel, 72 (35.5%) had VUS, and 40.3% (*n* = 29) of those patients with VUS had some form of cancer. This suggests the influence of one or more VUS that have not been previously categorized as pathogenic, but which have pathogenic activity in this Arab population.

A correlation analysis of variants showed 14 distinct pairs of variants were significantly correlated (frequently appearing together), even if the genes involved were on different chromosomes and therefore unlinked. This may be because several of the study participants were grouped into families, including some in which one or both parents had multiple variants; additionally, some variants are likely to be present in both parents due to the high degree of consanguinity in Saudi Arabia. Thus, the parents had an increased chance of passing on certain variants to their children. Of the 14 significant correlations, six involved PVs and a further three involved likely PVs (Supplementary Table 4). Notably, APC c.3920T>A was significantly linked to MSH2 c.1964del (*p* = 1.8e-07) and MSH6 c.733A>T (*p* = 0.02).

## DISCUSSION

Recent advances in the field of medical genetics have allowed a much deeper understanding of the underlying genetic risk factors for cancer. In particular, cancer screening utilizing germline genetic sequencing of panels of cancer susceptibility genes has become a powerful tool to identify potential underlying familial genetic variants and their associations with elevated cancer risks [40]. For example, studies within the Israeli Ashkenazi Jewish population, which is well-characterized, have illuminated the existence of three founder mutations that are estimated to account for up to 30% of early-onset breast cancer and 60% of ovarian cancer in this population [41, 42]. The discovery of these mutations has fundamentally changed the public health management of early-onset cancer in this population, which includes the recommendation of genetic testing for all individuals of Ashkenazi Jewish descent as part of early cancer detection and prevention [41]. There may be genetic variants associated with cancer in many ethnic or regional populations, such as people of Arab descent, that do not appear be associated with cancer, or that show lower association with cancer, in the European populations normally recruited for these types of association studies [14].

This observational study investigated familial cancer biomarkers in 110 cancer patients, their family members, and unrelated patients with other conditions. Roughly a quarter (24.4%, *n* = 31) of the family members of the patients with cancer who had not yet received any cancer diagnosis (*n* = 127), and 8 of 16 relatives with cancer themselves (50%), had at least one PV as defined based on PV effects demonstrated in non-Arab-specific populations (Supplementary Table 1). These data demonstrate a sufficient presence of genetic variants with the potential to increase cancer risk in the Saudi population to justify prioritizing genetic surveillance in Saudi Arabia. However, it must be acknowledged that PVs can have low penetrance, and often do not lead to disease onset [43].

The disproportionately high percentage of individuals with variants demonstrated in other settings to be pathogenic, combined with the higher number of homozygotes for PVs or likely PVs, in index patients and family members with cancer than would be expected from a proportional distribution, indicates that, despite the variant classifications being based on studies of non-Arab specific populations, they do apply, at large, to people of Middle Eastern descent.

The roughly proportionate numbers of cancer patients, family members with cancer and high-risk relatives carrying one or more VUS indicates that some of these variants are benign in those of Arab ethnicity. We should therefore not make any assumptions that each variant within our panel is relevant, or that the panel of potential variants is complete. While the overall categorization of variant types based on non-Arab populations applies to the study population, individual variants not believed to play a role in cancer occurrence in one population may exhibit such a role in another population [14]. Additionally, the frequencies of VUS, likely PVs and PVs can depend on the ancestry of the population [24]. Therefore, all variants found in the Arab, mostly Saudi Arabian, subjects of this study were statistically analyzed for their potential association with cancer in general Arab populations.

Where associations observed have previously been specifically linked to hereditary cancers in other settings, this leads us to believe that we are correct in considering them familial. Over half of all subjects had one or more variant in the *APC* gene, the most common of which was *APC* c.3920T>A; p.Ile1307Lys, which was significantly associated with colon cancer (*p* = 0.03predict). This is considered a low penetrance VUS in most settings [16], but it has been demonstrably associated with colorectal tumor risk in the Israeli Ashkenazi Jewish population [42]. *APC* c.3920T>A; p.Ile1307Lys was observed in 18 of 126 cancer cases (14.3%) and 10 of 67 colorectal cancer cases (14.9%), compared to 4 of 57 individuals without cancer or family history of cancer (7.0%). We propose that *APC* c.3920T>A; p.Ile1307Lys may act as a pathogenic variant in this population, and may prove a useful marker for colon cancer risk in Arab populations.

Multiple variants may impact the same signaling pathways to create a higher likelihood of cancer if appearing together. For example, reduced expression of *MUTYH* and *TP53* mutation, both of which are associate with diffuse-type histology, have been shown to be associated in adenocarcinomas of gastric cardia patients. In this cohort, *APC* c.3920T>A; p.Ile1307Lys was linked to *MSH2* c.1964del; p.Val655Aspfs*30 and *MSH6* c.733A>T; p.Ile245Leu. All three of these variants have been tentatively associated with Lynch syndrome. *MSH2* c.1964de has previously been identified in Lynch syndrome patients with colorectal tumors within Saudi Arabia [29].

It is our intention that our findings influence future screening protocols. We note with interest the diversity of *BRCA1 and BRCA2* variants within our cancer patient group, and the enrichment of *BRCA2* VUS in cancer patients. Whilst no specific variants dominated the pool, in general terms *BRCA2* variants were associated with cancer and *BRCA2* confirmed PVs were strongly associated with breast cancer collectively (*p* = 8e^−06^). Only in one instance did a breast cancer patient have both *BRCA1* and *BRCA2* variants. Despite identifying five ovarian cancer patients, there was no crossover between the variants we identified in our patients with cancer and the five most common *BRCA1* variants identified in the meta-analysis by Younes and Zayid [45], nor any carriers of the key Ashkenazi ‘founder’ *BRCA1* and *BRCA2* variants [41, 42].

The *BRCA1* variant c5530delC, which has been flagged as unique among Saudi Arabian ovarian cancer patients [45], and one of the most common variants observed in another Saudi Arabian ovarian cancer cohort by Agha et al. [49], was present in one subject with ovarian cancer, with each patient carrying a different variant (3 in *BRCA1*, 1 in *APC*, 1 in *TP53*, see Supplementary Table 3). We note a similar overall incidence of *BRCA1* mutation in association with ovarian cancer between this study and that of Agha et al. (60% vs. 77%), although our ovarian cancer patient pool is small. Screening for a diverse panel of *BRCA1* and *BRCA2* variants may be necessary to protect women in Arab populations who have a known family history of breast and/or ovarian cancer.

As *APC* c.3920T>A; p.Ile1307Lys was found to be associated with a higher occurrence of CRC (including colon, rectal and sigmoid cancers, as well as polyposis; *p*-value = 0.0257), the unexpected frequency of the variant in low risk individuals may indicate a greater predisposition for, or prevalence of, CRC in the general Saudi population than has been previously detected. Asymptomatic individuals with this variant may need to be followed up by an oncologist, as they may develop cancer in the future. A larger study might reveal the need for national screening for this variant in any Saudi individuals who have a family history of CRC, and this variant may function as a CRC-associated marker in the Saudi population. Until further investigation can be completed into the specific impact of frequent and rare *APC* and *BRCA2* variants in Arab cohorts, all patients carrying either *APC* or *BRCA2* variants should be considered at elevated risk of CRC and breast cancer and be offered screening accordingly.

Whilst frequent variants are excellent candidates for future screening panels, rarer variants are important to acknowledge. *TP53* c.868C>T; p.Arg290Cys was significantly associated with multiple colon polyposis in this population, despite not having been previously linked to any specific cancer form. In our study population, *TP53* c.868C>T; p.Arg290Cys is carried by five members of the same family, two of which have polyposis and one of which may have polyposis. The family do not share any other variant detected in this gene panel.

There were many variants appearing 3 or more times only in cancer and/or high-risk subjects which should be considered to be potential PVs (*TP53* c.799C>, *APC* c.3183_3187delACAAA, *ATM* c.1516G>T, *BRCA2* c.3170_3174del, c.122C>T, c.5909C>T and c.5557dup, *CDKN2A* c.238C>T, *MSH2* c.1964del, *MSH6* c.733A>T deletion of the whole of exon 16 of *MLH1, MUTYH* c.544C>T and c.734G>A, and *PALB2* c.1102A>T). All *TP53* c.799C > T carriers were those patients syndicated from a previous family case study [44], with no additional carriers of this variant identified among our patient cohort or their family members.

**Figure 4:**
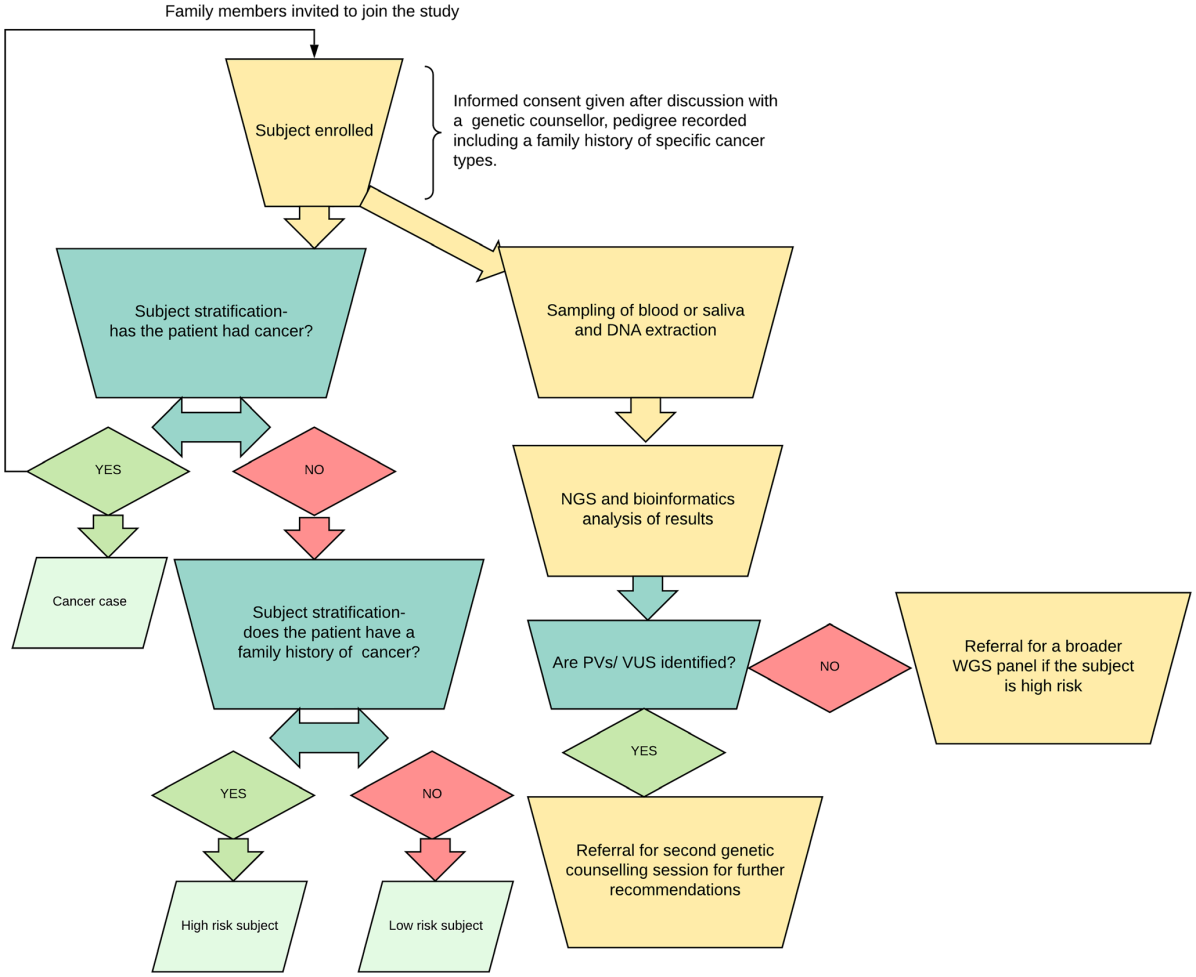
Workflow diagram. Subjects were stratified into three groups, their saliva samples were processed and sent for next generation sequencing. Family history was defined, in order to apply to all cancer subgroups, as having one or more relatives (parents, grandparents etc.) with any type of cancer. A breakdown of the relationships to the cancer patient subject demographic data for all subjects are available in Supplementary Table 1.

Limitations of this study included limited sample size. However, this is still the largest prospective study of its type in this population. Another limitation of the study is the uneven distribution of ages between various subject and control groups, involving a skewing towards older individuals in patients with cancer. A longitudinal follow-up studies of these high-risk relatives, low-risk individuals, or controls with variants should reveal or strengthen any such associations over time. The limited NGS panel was intended to be used as preliminary screening tool, therefore we recommended a more comprehensive panel to be used in negative high-risk cases (Figure 4). Finally, the cancer negative patients screened via panel test are not fully representative of a healthy population, as each had some clinical symptoms or family history prompting their initial panel testing for other purposes.

These results indicate that, in general, the categorization of PVs based on their effects on non-Arab populations may produce an incomplete picture of variants with relevance for a Saudi population. Some PVs common in Caucasian populations be less frequent or less likely to predict cancer onset, and some VUS that occur at low frequency in other reported populations may play a primary role in cancer risk in Arab populations.

In conclusion, this study is one of the first to report the prevalence of inherited cancer genetic variants in a cohort from the Arab world. Our study gives critical first insights into the genetic variants associated with overall cancer risk in this specific population, and specific forms including CRC/Lynch syndrome and breast cancer. Whilst a larger population level study is still needed, we demonstrate that multigene NGS panel testing may serve as non-invasive diagnostic and cost-effective tool to predict familial cancer risk at the pre-clinical stage, allowing targeted screening and enabling early intervention.

## MATERIALS AND METHODS

### Subjects

The study cohort was recruited from the King Fahad Medical City (KFMC) clinical departments and from other medical centers throughout Saudi Arabia, and therefore represents a geographically diverse set of patients. KFMC is a major tertiary referral center in Middle East with ~1200 beds and 8 specialized centers including a comprehensive cancer center. Cancer patients diagnosed with breast, ovarian, colorectal, brain, thyroid, melanoma, pancreatic, prostate, uterine, or stomach cancer, and their family members, were invited to participate in the study. All of the subjects were referred to the Familial Genetic Counseling Clinic for a preliminary meeting, and again if relevant following sequencing, to be informed of any mutations relevant to their health. The majority of participants were Saudi Arabian nationals, and a small minority represented other Arab nationalities (Table 1). Although race and ethnicity data were not available, an estimated 90% of Saudi Arabian nationals are Arab (CIA, 2022), therefore we assume our cohort to be predominantly Arab.

The panel results of two of the included patients, neither or which had a history of cancer, were syndicated from another clinic within the same medical group (Supplementary Table 1).

### Study procedures

Enrolled subjects were stratified into groups, and their DNA samples were obtained and processed, after seeing a certified genetic counselor in a cancer genetics clinic and obtaining informed consent (Figure 4).

### Next generation sequencing

Target enrichment was carried out according to Agilent’s SureSelect method (v1.7), followed by sequencing via Illumina’s NextSeq 500 or NovaSeq600 (paired-end 150 bp, High Output kit), conducted at Color^®^ Genomics Laboratory, to analyze the panel of 30 genes featured in the cancer susceptibility gene panel tool kit from Color^®^ Genomics Laboratory (*APC, ATM, BAP1, BARD1, BMPR1A, BRCA1, BRCA2, BRIP1, CDH1, CDK4, CDKN2A* [*p14ARF* and *p16INK4a*], *CHEK2, EPCAM, GREM1, MITF, MLH1, MSH2, MSH6, MUTYH, NBN, PALB2, PMS2, POLD1, POLE, PTEN, RAD51C, RAD51D, SMAD4, STK11*, and *TP53*), in which mutations have been associated with an elevated risk for breast, ovarian, colorectal, brain, thyroid, melanoma, pancreatic, prostate, uterine, or stomach cancers (Supplementary Table 2). The majority of these genes were assessed for variants within all coding exons with +/− 20 bp flanking each exon. This panel kit should broadly capture gene variants reported to be associated with a variety of cancers, and has been applied across diverse research settings to characterize cancers and to determine patient risk [35–38, 50, 51]. The methodology was followed by our team as recommended by the provider, including quality control checks incorporated to ensure proper sample identification and efficiency of DNA isolation, library preparation and target capture. In addition, each sequencing test contained two fully-characterized positive controls. The Color Test for hereditary cancer has been developed in compliance with the Clinical Laboratory Improvements Act of 1988.

### Bioinformatics analysis

The bioinformatics analysis of sequence data followed a previously published pipeline [37, 38]. Copy number variations were detected using dedicated internally developed algorithms for read depth analysis and split-read alignment detection. Variants were classified according to the standards and guidelines for sequence variant interpretation of the American College of Medical Genetics and Genomics (ACMG) [37, 38, 52] into pathogenic, likely pathogenic, variant of uncertain significance (VUS), likely benign, and benign categories. All variants were evaluated by a board-certified medical geneticist or pathologist. Variants classified as pathogenic or likely pathogenic were confirmed through secondary technology (Sanger sequencing, array comparative genomic hybridization, or multiplex-ligation dependent probe amplification) before reporting.

### Statistical analysis

All statistical analyses were conducted using R statistical software v4.1.2 and R package XNomial (function”xmulti”) v1.0.4. Study participants were classified according to whether they had only PVs, likely PVs, VUS (with variants reported to have conflicting interpretations of pathogenicity considered as VUS), or various combinations of these three variant types. Variant classifications were assigned to the genetic variants found in subjects following the criteria of the ACMG, based on characteristics described either on the ClinVar database (https://www.ncbi.nlm.nih.gov/clinvar/) or in the literature, although we apply these criteria tentatively to this Arab-specific study population. Statistical tests were conducted as described in the Supplementary Methods.

## SUPPLEMENTARY MATERIALS





## Abbreviations

NGS: next-generation sequencing
PV: pathogenic variant
FCS: familial/hereditary cancer syndromes
CRC: colorectal cancer
CPC: choroid plexus carcinoma
ICPI: immune checkpoint inhibitor
KFMC: King Fahad Medical City
VUS: variant of uncertain significance
