# Supplementary material for: Investigating the prevalence of pathogenic variants in Saudi Arabian patients with familial cancer using a multigene next generation sequencing panel
Source: Oncotarget. 2023 Jun 12;14:580–94. doi: 10.18632/oncotarget.28457 (PMC10259259; doi:10.18632/oncotarget.28457)
Supplement: Supplementary file 1 [file oncotarget-14-28457-s001.pdf]

Investigating the prevalence of pathogenic variants in Saudi Arabian patients with familial cancer using a multigene next generation sequencing panel

SUPPLEMENTARY MATERIALS

Supplementary Methods

Statistical tests

The age and gender distribution of subjects recruited into the study were compared using chi-squared goodness of fit tests. The proportions of individuals in the various subject (i.e., index patients and family members with cancer, high-risk relatives, or low-risk individuals), and control categories were analyzed using Fisher-Freeman-Halton tests for a disproportional association between each category and variant class. Additionally, a likelihood ratio version of the exact test for a multinomial distribution (multinomial likelihood test) was conducted to reveal any disproportionality in the numbers of carriers of each mutation between the different variant classes in the subject and control groups. Pairwise correlation of variants was measured using the Pearson method and the Holm correction was used to address multiple comparisons.

Supplementary Data 1: See Supplementary Data 1

Supplementary Figures: See Supplementary Figures

Supplementary Table 1: Detailed patients cohort. See Supplementary Table 1

Supplementary Table 2: Detailed patients cohort

All		
**Genetic test result**	***n***	**%**
Positive for PVs	119	38.39%
Negative for PVs	117	37.74%
VUS only	72	23.23%
Not tested-positive (results syndicated from another study)	1	0.32%
Not tested-negative (results syndicated from another study)	1	0.32%
Sample Failed (excluded from analysis)	3	
**Cancer patients**		
Genetic Test Result	*n*	%
Positive for PVs	40	36.36%
Negative for PVs	42	38.18%
VUS only	28	25.45%
**Relatives with cancer**		
Genetic Test Result	*n*	%
Positive for PVs	9	56.25%
Negative for PVs	6	37.50%
VUS only	1	6.25%
**High risk individuals**		
Genetic Test Result	*n*	%
Positive for PVs	52	41.27%
Negative for PVs	48	38.10%
VUS only	26	20.63%
**Low risk individuals**		
Genetic Test Result	*n*	%
Positive for PVs	17	29.31%
Negative for PVs	21	36.21%
VUS only	17	29.31%
Not tested-positive (results syndicated from another study)	1	1.72%
Not tested-negative (results syndicated from another study)	1	1.72%

Supplementary Table 3: Detailed patients cohort. See Supplementary Table 3

Supplementary Table 4: Significant pairwise correlations between variants determined using Pearson's correlation test with holm correction

Variant 1	Variant 2	Correlation *P* value	PV involved	Likely PV involved	VUS only
APCc.3205A>G	PMS2c.1376C>G	6.85E-12	Y	N	N
APCc.3566C>T	MSH6c.733A>T	0.007413263	N	N	Y
ATMc.1516G>T	ATMc.8520G>C	3.55E-05	N	N	Y
ATMc.1516G>T	BRCA1c.5251C>T	1.70E-08	Y	N	N
ATMc.1516G>T	MSH2c.508C>G	6.63E-05	N	N	Y
ATMc.1516G>T	MSH2c.862C>T	5.68E-05	Y	N	N
BRCA2c.122C>T	MLH1c.91_92delGCinsTG	0.006362677	N	N	Y
BRCA2c.7628A>G	PMS2c.1606C>T	1.56E-03	Y	N	N
CDKN2A (p14ARF) Whole gene deletion (due to major chomosomal alteration)	PMS2c.1376C>G	6.85E-12	Y	Y	N
MSH2c.1964del	APCc.3920T>A	1.82E-07	N	Y	N
MSH6c.733A>T	APCc.3920T>A	0.022154349	N	Y	N
PMS2c.1606C>T	APCc.7646G>A	7.88E-11	Y	N	N
TP53c.799C>T	BRCA2c.7534C>T	4.71E-06	N	Y	N

Significant pairwise correlations between variants determined using Pearson's correlation test with Holm correction.
